# Distance measurements via the morphogen gradient of Bicoid in *Drosophila *embryos

**DOI:** 10.1186/1471-213X-10-80

**Published:** 2010-08-02

**Authors:** Feng He, Ying Wen, David Cheung, Jingyuan Deng, Long J Lu, Renjie Jiao, Jun Ma

**Affiliations:** 1 State Key Laboratory of Brain and Cognitive Science Institute of Biophysics Chinese Academy of Sciences 15 Datun Road Beijing 100101, China; 2 Graduate School of the Chinese Academy of Sciences Beijing 100049, China; 3 Divisions of Biomedical Informatics Cincinnati Children's Research Foundation 3333 Burnet Avenue Cincinnati, OH 45229, USA; 4 Developmental Biology Cincinnati Children's Research Foundation 3333 Burnet Avenue Cincinnati, OH 45229, USA; 5 Department of Biomedical Engineering University of Cincinnati Cincinnati, OH 45221, USA

## Abstract

**Background:**

Patterning along the anterior-posterior (A-P) axis in *Drosophila *embryos is instructed by the morphogen gradient of Bicoid (Bcd). Despite extensive studies of this morphogen, how embryo geometry may affect gradient formation and target responses has not been investigated experimentally.

**Results:**

In this report, we systematically compare the Bcd gradient profiles and its target expression patterns on the dorsal and ventral sides of the embryo. Our results support a hypothesis that proper distance measurement and the encoded positional information of the Bcd gradient are along the perimeter of the embryo. Our results also reveal that the dorsal and ventral sides of the embryo have a fundamentally similar relationship between Bcd and its target Hunchback (Hb), suggesting that Hb expression properties on the two sides of the embryo can be directly traced to Bcd gradient properties. Our 3-D simulation studies show that a curvature difference between the two sides of an embryo is sufficient to generate Bcd gradient properties that are consistent with experimental observations.

**Conclusions:**

The findings described in this report provide a first quantitative, experimental evaluation of embryo geometry on Bcd gradient formation and target responses. They demonstrate that the physical features of an embryo, such as its shape, are integral to how pattern is formed.

## Background

A cornerstone in developmental biology is the concept of morphogens. These are chemical substances that form concentration gradients to provide positional information to a developing embryo or tissue [1-5]. Despite significant advances in the understanding of morphogens in recent years, how the geometry of an embryo or tissue may affect morphogen gradient formation has not been well investigated experimentally. This question, like other quantitative questions about morphogens, requires an accurate measurement of the gradient concentrations, a task that poses significant technical and analytical challenges [6-8]. Recent efforts, including live-imaging studies [6] and our own embryo staining studies [8,9] of the gradient of the *Drosophila *morphogen Bicoid (Bcd), have overcome such challenges and revealed important new insights into the behavior of this morphogen gradient (see also [10]). Here we extend our studies to investigate the effects of embryo geometry on Bcd gradient formation and the interpretation of its encoded positional information.

Bcd, a morphogen which forms a gradient along the anterior-posterior (A-P) axis in *Drosophila *embryos, is responsible for patterning the anterior structures, including the head [11,12]. It is a transcription factor that directly activates the expression of its downstream target genes in a concentration-dependent manner [13-15]. Bcd gradient formation takes place before the physical boundaries of cells (i.e., cell membranes) are formed in the embryo. It is a process [16] thought to involve localized synthesis of Bcd protein molecules in the anterior coupled with diffusion and degradation of these molecules throughout the embryo (see also [17]). At the syncytial blastoderm stage when Bcd begins to establish and refine its concentration gradient and activate its target genes, the embryo already possesses two distinct diffusion media for Bcd molecules: the inner part (yolk) of the embryo and the nuclear layer on the cortex [18,19]. Since the cortex of the embryo is a curved surface, embryo geometry may affect how the Bcd gradient is formed and, as a consequence, how its target genes are activated during development.

In this study, we systematically analyze the behaviors of both the Bcd gradient and its target responses on the dorsal (D) and ventral (V) sides of the embryo to investigate the effects of embryo geometry on morphogen gradient formation and action. We sought to address the question, through quantitative studies of Bcd and its direct gap gene targets, of how segmentation gene expression patterns exhibit differences between the dorsal and ventral sides. Previous studies show that the expression boundaries of gap genes and expression stripes of pair-rule genes are not parallel to the D-V axis, but rather present a splay on the ventral side of the embryo [20-23]. How such a splay is established remains controversial because several models, ranging from an interaction between the A-P and D-V patterning systems [21] to embryo geometry [24] and nuclear density/movements [25], have been proposed. Our experimental results reveal important insights into how distance is properly measured and positional information is encoded by the Bcd gradient in embryos. We also present 3-D simulation results demonstrating that a curvature difference between the two sides of an embryo is sufficient to generate Bcd gradient properties that are consistent with experimental observations. Our systematic analyses of a direct Bcd target, Hunchback (Hb), show that both dorsal and ventral sides of the embryo are fundamentally similar to each other in terms of Hb response to the Bcd gradient. These results suggest an important role of embryo geometry, operating through its effects on the Bcd gradient, in establishing differences of segmentation gene expression patterns between the dorsal and ventral sides of the embryo. Together, our findings demonstrate that the physical features of an embryo, such as its shape, can directly affect the outcome of the patterning process.

## Results

### Embryo shape and design of study

**Figure 1:**
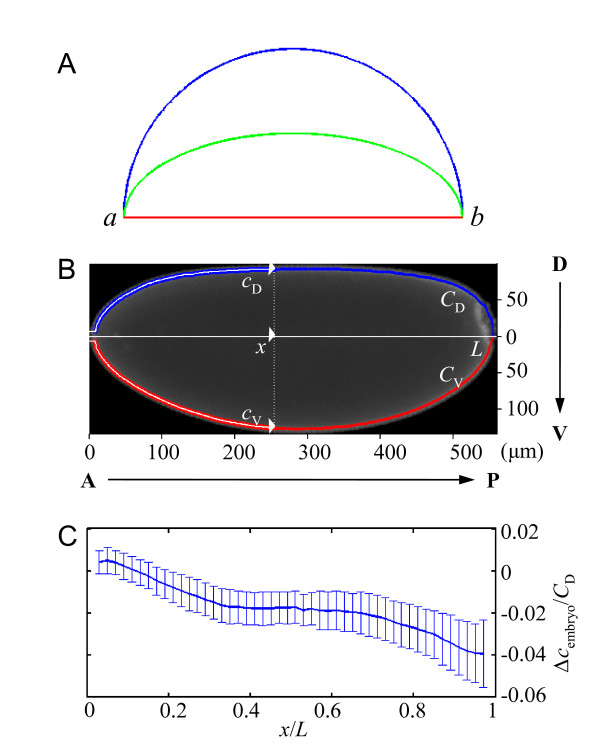
**Dorsal and ventral sides of the embryo have different geometric features**. (A) Illustration showing different distances in geometry. (B) Digital image from DAPI channel of a wt (*w^1118^*) embryo to illustrate the parameters used to analyze geometric features in this study. (C) Relative differences (with error bar shown) in contour distances between the dorsal and ventral sides (Δ*c*_embryo_/*C*_D_) as a function of fractional embryo length (*x*/*L*) from 28 wt embryos at early nuclear cycle 14. All distances along the A-P axis in this report are measured from the anterior pole unless otherwise stated.

In physics and mathematics, the distance along a curved path between two points is distinct from the displacement along a straight line connecting them. For instance, in Fig. 1A the red line represents the displacement from point *a *to point *b*, which is shorter than the distance along a semi-circle (blue) or a partial ellipse (green). While displacement is simply the length of the line segment between points *a *and *b*, more quantities such as curvature are required to determine the distance along a curved path. In developmental biology, how distance is measured (i.e., positional information encoded) by morphogen gradients is an important question. This question is directly relevant to the fundamental problems of how morphogen gradients are formed and how their encoded positional information is interpreted in a developing embryo or tissue. In early *Drosophila *embryos, the dorsal and ventral sides exhibit different geometric features. For example, as shown in Fig. 1B, the contour length measured along the perimeter of the dorsal side (blue), *C*_D _= 645.4 ± 18.3 *μ*m (standard deviation from 28 wt embryos), is significantly shorter than the contour length measured along the perimeter of the ventral side (red), *C*_V _= 674.9 ± 17.2 *μ*m (*p *= 10^-7^, two-tailed Student's t-test). The difference between the two contour lengths, *C*_V _and *C*_D_, is a direct consequence of the different curvatures on the two sides of the embryo.

In our current study, we take advantage of the geometric differences between the dorsal and ventral sides of the embryo to facilitate our investigation of how the Bcd gradient is formed and how its direct gap gene targets are activated during development. We analyze Bcd gradient profiles and its target responses using A-P positions that are defined in two distinct ways: projected distance *x *from the anterior pole or contour distance *c *from the identical anterior pole (Fig. 1B). Technically, while *x *is measured by projecting the coordinate of the center of an intensity scanning window onto the A-P axis, contour distance *c *is measured as the route along the perimeter of either the dorsal or ventral side of the embryo (*c*_D _and *c*_V_, respectively; see Methods for details on *c *determination). In Fig. 1C, we plot the average difference between the two contour distances, Δ*c*_embryo_/*C*_D _(Δ*c*_embryo _= *c*_D _- *c*_V_), against fractional embryo length (i.e., the normalized projected distance) *x*/*L *in wt embryos. This plot reveals the following information regarding the geometric differences between the dorsal and ventral sides of the embryo: 1) *c*_V _is longer than *c*_D _at same *x/L *positions in most parts of the embryo, except near the anterior pole (*x*/*L *< ~0.12); 2) there are two broad regions of the embryo, ~0.12 <*x*/*L *< ~0.36 and ~0.66 <*x*/*L *< ~0.96, where *c*_V _expands/accumulates its lead over *c*_D_; 3) in the mid-embryo section along the A-P axis (~0.36 <*x*/*L *< ~0.66), Δ*c*_embryo _is stable, reflective of a small curvature difference between the two sides of the embryo (see additional file 1 for a theoretical 2-D consideration of geometry and positional information). These observed differences between the dorsal and ventral sides represent important tools to facilitate our study of Bcd gradient formation and target gene activation during development. Here we compare the behaviors of the Bcd gradient profiles and its target responses on both dorsal and ventral sides of the embryo. An important premise of our work is that the observed Bcd gradient properties can directly shed light on how distance is measured and positional information encoded during development. Thus, if the positional information encoded by the Bcd gradient is based on the projected distance *x *in embryos, the profiles from the dorsal and ventral sides are expected to be more similar to each other as a function of *x *than as a function of *c*. The converse is expected to be true if the encoded positional information is based on the contour distance.

### Evaluating two different distance measurements for the Bcd gradient

In our analysis, embryos at early nuclear cycle 14 were triple-stained with anti-Bcd and anti-Hb antibodies as well as DAPI (see Methods for details). We captured high-resolution digital images on the midsagittal plane of the embryos and measured fluorescence intensities along the cortical, nuclear layer (Fig. 1B). As further discussed in our recent work [8], we used raw Bcd intensities *B *in our analysis, with background subtracted when necessary (see Methods for details); our experimental conditions yielded Bcd intensity profiles with a reproducibility that is comparable to live-imaging studies [6,8]. The high precision in Bcd intensity measurements and the use of raw Bcd intensities make the analyses described in this report possible. As discussed in detail previously [8], normalization of Bcd intensities can mask or distort the system properties. Using a pixel-by-pixel scanning approach (see Methods for details), we recorded both the intensity values and the *x*-*y *coordinates in microns in order to quantify the intensity profiles using two distinct distance measurement methods: *x *and *c *as defined above.

**Figure 2:**
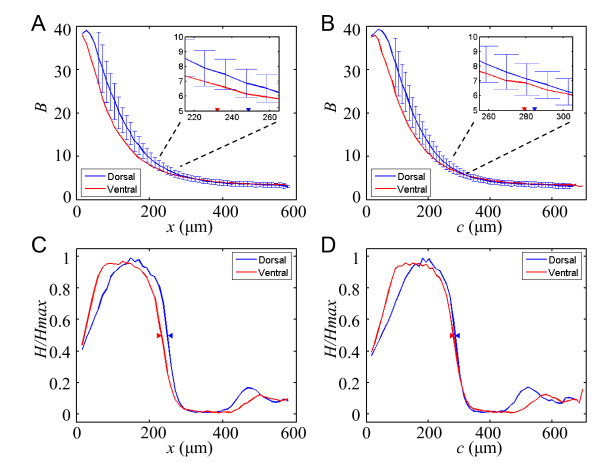
**Bcd and Hb profiles expressed in two different distance measurements**. (A and B) Average raw Bcd intensity profiles on the dorsal (blue) and ventral (red) sides of 28 wt embryos when measured as a function of projected distance *x *(A) or contour distance *c *(B) from the anterior. Error bars are shown for the profile from the dorsal side. In each panel, the inset shows a magnified view of the region surrounding the Hb expression boundary positions, which are marked with arrowheads for reference. (C and D) Average normalized Hb intensity profiles on the dorsal (blue) and ventral (red) sides of the same wt embryos when measured as a function of projected distance *x *from the anterior (C) or contour distance *c *(D). The Hb expression boundary positions on the two sides are marked with arrowheads.

To gain insights into how distance is measured by the Bcd gradient in embryos, we plotted average raw Bcd intensities *B *from the dorsal and ventral sides against either *x *or *c *in wt embryos. We binned all recorded positions from all embryos using two distinct methods: 1) either using a constant *x *interval (11 pixels in this study) between neighboring bins for plotting Bcd intensity profiles as a function of projected distance *x *from the anterior, or 2) using a constant *c *interval (also 11 pixels) between neighboring bins for plotting Bcd intensity profiles as a function of contour distance *c *from the anterior. Our results show (Fig. 2A) that, around the Hb boundary positions, the two average Bcd raw intensity profiles from the dorsal and ventral sides of the wt embryos are significantly different from each other when measured as a function of projected distance *x *from the anterior (*p *= 0.001, 0.005, 0.002, 0.006, and 0.02 at the error bars shown in the inset; two-tailed Student's t-tests). In contrast, these two average Bcd profiles are much closer to each other when measured as a function of contour distance *c *(Fig. 2B; *p *= 0.06, 0.09, 0.36, 0.18, and 0.50 at the error bars shown in the inset). As explained above, these results are supportive of a hypothesis that, at the syncytial stage, the contour distance *c*, as opposed to projected distance *x*, represents a more proper measurement of distance during Bcd gradient formation (see also additional files 2, 3 and 4 for results in 1×-*bcd *and 3×-*bcd *embryos; and additional files 5 and 6 for Bcd and Hb intensity data). We will return to this hypothesis by further considering the physical process of Bcd gradient formation and simulating, in a new 3-D model, the biological effects of embryo geometry on Bcd gradient profiles.

### Measurements of Hb expression boundary positions using two methods

To determine whether the positional information encoded by the Bcd profiles on the dorsal and ventral sides of the wt embryos are properly interpreted by its downstream target responses, we measured Hb expression profiles in wild-type (wt) embryos. To ensure a direct evaluation of the relationship between Bcd and Hb, we extracted Bcd and Hb intensities in wt embryos that were simultaneously stained with both Bcd and Hb antibodies (see Methods for details). Again, we analyze Hb expression profiles in two distinct ways: as a function of either projected distance *x *from the anterior or contour distance *c*. This allows us to measure the Hb expression boundary position (the location where Hb intensity level is half maximal) as either projected distance *x*_Hb _from the anterior or contour distance *c*_Hb_. Fig. 2C and 2D show the average Hb intensity profiles from the dorsal and ventral sides as a function of either *x *or *c*, respectively. While these two Hb profiles are visually different from each other when measured as a function of *x *(Fig. 2C), they converge near mid-embryo when measured as a function of *c *(Fig. 2D). Quantitatively, while the *x*_Hb _values on the dorsal and ventral sides of wt embryos (*x*_HbD _and *x*_HbV_, respectively) are significantly different from each other (Δ*x*_Hb _= *x*_HbD _- *x*_HbV _= 248.7 - 232.2 = 16.5 *μ*m, *p *= 10^-8^; Fig. 2C), the difference between the *c*_Hb _values on these two sides of the embryos becomes much smaller (Δ*c*_Hb _= *c*_HbD _- *c*_HbV _= 284.6 - 279.1 = 5.5 *μ*m, *p *= 0.07; Fig. 2D). The observed value of Δ*c*_Hb_, but not Δ*x*_Hb_, is comparable to the diameter of a single nucleus (~6.5 *μ*m [19]) or the embryo-to-embryo variation of the Hb expression boundary position (0.014 *L *or 7.8 *μ*m [8]). These results show that Hb, as a biological readout of the Bcd gradient, exhibits expression properties that are consistent with those of the Bcd gradient in terms of how distance is measured (i.e., positional information encoded) in embryos. Together, these results suggest that both Bcd profiles and its encoded positional information (as monitored by Hb expression boundary positions) follow a function of contour distance *c *from the anterior, as opposed to projected distance *x*.

### Input-output relationship on dorsal and ventral sides of the embryo

**Figure 3:**
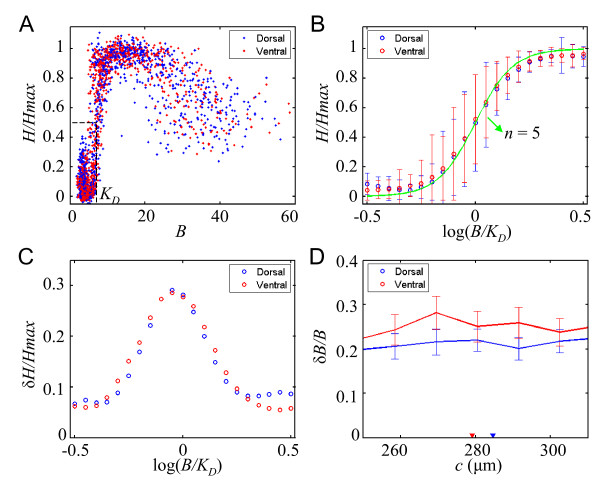
**Similar Bcd-Hb input/output relationship on D and V sides**. (A) Scatter plots of normalized Hb intensity values against raw Bcd intensity values on the dorsal (blue) and ventral (red) sides of wt embryos. The effective dissociation constant *K*_d _for Bcd activating Hb expression on the two sides can be extracted from the plots, as illustrated by dashed lines. (B) Average input/output relationship on the dorsal (blue, with error bars) and ventral (red) sides of wt embryos. *B *and *K*_d _values are background-subtracted. Green profile represents a Hill curve with cooperativity *n *= 5. (C) Intensity noise of Hb expression in response to the Bcd gradient. *B *and *K*_d _values are background-subtracted. (D) Intensity noise of Bcd at and around *c*_Hb _on the dorsal (blue) and ventral (red) sides of wt embryos. In this figure (and Fig. S1 H and S2H), noise from measurement and background is not subtracted from the Bcd intensity noise profiles.

It has been suggested that the A-P and D-V patterning systems can interact with each other during development [21,26-28]. Such an interaction may represent an important mechanism to influence how Bcd targets are expressed on the dorsal and ventral sides of the embryo. To further determine whether Bcd molecules have similar or distinct biological activities on these two sides of the embryo, we analyzed the input-output relationship between Bcd and Hb. The input-output relationship depicts the biological activities of the Bcd gradient in stimulating its target gene expression during development. As shown in this Fig. 3A, the two clusters of data points overlap with each other, both exhibiting a steep on/off switching pattern. These results demonstrate that both dorsal and ventral sides of the embryo have a similar Hb response profile to the Bcd concentration gradient.

To evaluate the Bcd-Hb relationship more quantitatively, we plotted the average Hb intensities versus Bcd intensity (*B*/*K*_d_) for the dorsal and ventral sides of the embryo (Fig. 3B, blue and red, respectively). *K*_d _is the dissociation constant, which reflects the effective Bcd concentration required for the Hb on/off switch, measured as the background-subtracted Bcd fluorescence intensity value at *c*_Hb_. The Bcd-Hb relationship on both sides fits well to the Hill's law as expressed in the following equation:(1)

where *n *is the Hill coefficient, which defines the steepness of the Hb on/off switch in response to the Bcd gradient (in Fig. 3B the green profile is, for comparative purposes, the Hill curve of *n *= 5, a value comparable to what is observed experimentally in DNA binding studies [29]). Since these two parameters provide quantitative measures of the Bcd-Hb relationship, we compared their values calculated from both sides of individual embryos. Our results show that neither *K*_d _values (expressed as arbitrary intensity units) on the dorsal and ventral sides of the embryo (5.0 ± 1.2 and 4.8 ± 1.2, respectively; *p *= 0.53) nor their *n *values (5.1 ± 2.7 and 4.9 ± 2.7; *p *= 0.73) exhibit a significant difference. Finally, to evaluate the precision of the Hb readout machinery in response to the Bcd gradient, we calculated the normalized standard deviations of Hb expression (*δH*/*H*_max_) on both sides of the embryo as a function of Bcd concentration (*B*/*K*_d_). Our results (Fig. 3C) reveal a globally similar profile of Hb expression noise in response to Bcd on both sides of the embryo. Together, these results demonstrate a fundamentally similar Bcd-Hb relationship on the dorsal and ventral sides of the embryo.

### Analyzing precision of the Bcd gradient on dorsal and ventral sides

To further evaluate the Bcd and Hb behaviors on the dorsal and ventral sides of the embryo, we compared their Bcd intensity noise *δB*/*B *(standard deviation normalized by the mean) profiles. Previous studies including our own focused primarily on the reproducibility of the Bcd gradient on the dorsal or lateral side of the embryos [6,8,30-32]. In all those studies, Bcd intensity profiles were measured as a function of projected distance *x*. Here we measure them as a function of *c *and directly compare the measured Bcd intensity noise on dorsal and ventral sides of the embryo. Our results show that the ventral side has a higher *δB*/*B *around *c*_Hb _than the dorsal side (Fig. 3D). Although the precise mechanism(s) for this intensity noise difference remains to be elucidated, here we simply take advantage of this difference to further help us evaluate the Bcd-Hb relationship. To directly compare the precision of positional information provided by the Bcd gradient, we converted Bcd intensity errors *δB *(standard deviation) to its positional errors *σc *[6]. Our results reveal a higher *σc *on the ventral side (39 ± 1.2 *μ*m at *c*_Hb_; standard deviation calculated by bootstrapping) than the dorsal side (27 ± 0.6 *μ*m at *c*_Hb_). If Hb expression properties are dictated by Bcd gradient properties as suggested above, Hb expression is expected to be more variable on the ventral side than on the dorsal side. To specifically test this prediction, we compared the precision of *c*_Hb _on the two sides of the embryos. Our results show that the variability of Hb boundary on the ventral side (*σc*_Hb _= 11.3 ± 1.3 *μ*m) is higher than that on the dorsal side (*σc*_Hb _= 9.1 ± 0.9 *μ*m). These results provide further support to our conclusion that the dorsal and ventral sides of the embryo are fundamentally the same in regard to the Bcd-Hb relationship.

### Contributions of Bcd gradient profiles to dorsal-ventral differences

**Figure 4:**
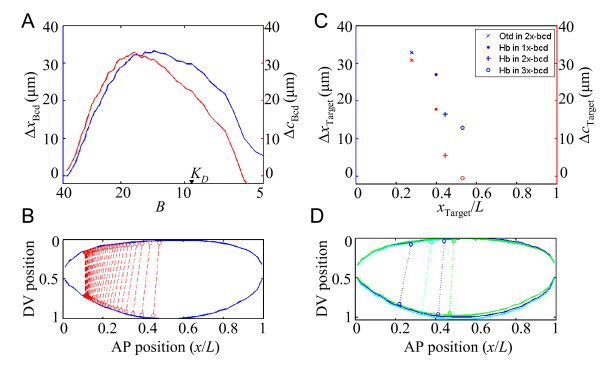
**Contributions of Bcd gradient behaviors to target expression slanting**. (A) Profiles of distance differences between the dorsal and ventral sides of the wt embryos when measured as either projected distance (blue, left scale) or contour distance (red, right scale). The profiles are shown as a function of raw Bcd intensity on log scale. (B) Positions with equal Bcd intensity values are marked on the dorsal and the ventral sides of an average wt embryo frame. Lines that connect the locations on both sides of the embryo can be viewed as iso-concentration contour lines. The embryo shape (blue) is averaged from the 28 wt embryos analyzed in this study, with dorsal side up and anterior to the left. (C) Differences in target boundary positions between the dorsal and ventral sides. The expression boundary positions are measured as either projected distance from the anterior (blue, left scale) or contour distance (red, right scale), and the differences are plotted at their respective projected boundary positions (measured from the dorsal side and expressed as fractional embryo length *x*/*L*). From anterior to posterior, each pair of results represents data in the following order: Otd in 30 wt embryos, Hb in 24 1×-*bcd *embryos, Hb in 28 wt embryos, and Hb in 29 3×-*bcd *embryos. (D) Target expression boundary locations on both dorsal and ventral sides are marked on the average embryos frames. The average 1×-*bcd *embryo frame is shown in cyan, wt in blue, and 3×-*bcd *in green. Both Otd and Hb boundary positions are marked on the average wt embryo frame. Lines connecting the target boundary positions on two sides of the embryos are shown for visual comparison with those shown in Fig. 4B.

Our analyses discussed thus far show that, in wt embryos, Hb boundary positions on the dorsal and ventral sides are significantly different from each other when measured as a function of projected A-P position. This difference, defined as Δ*x*_Hb _(see above for definition), effectively depicts a tilt or slant of the Hb expression boundary toward the anterior pole on the ventral side, which is reminiscent of the splay of A-P gene expression stripes at later stages of nuclear cycle 14 [20-25]. While this ventral splay is well-documented, its underlying mechanisms remain controversial (see Background and Discussion for details). To further study the contributions of Bcd gradient profiles to the expression slanting of its direct gap gene targets, we analyzed the data shown in Fig. 2A and 2B in an alternative way that measures the differences in locations at given thresholds of Bcd concentrations on the two sides of the embryo. Here, we plot such differences, Δ*x*_Bcd _and Δ*c*_Bcd _(Δ*x*_Bcd _= *x*_BcdD _- *x*_BcdV _and Δ*c*_Bcd _= *c*_BcdD _- *c*_BcdV _where *B*_D _= *B*_V_), as a function of Bcd concentrations on log scale (Fig. 4A). Since the Bcd profiles follow an exponential function except at the most anterior part where Bcd concentrations are high, this plot may be viewed as an equivalent (but not identical) to a plot of Δ*x*_Bcd _or Δ*c*_Bcd _against the A-P position. Fig. 4B is a presentation of these same results in a visually more straightforward manner, where the locations of equal mean Bcd intensities are marked on the dorsal and ventral sides of an average wt embryo frame on the midsagittal plane (see Methods for details).

Our results shown in Fig. 4A make four specific, experimentally testable predictions if the Bcd profiles are responsible for the expression slanting of its direct gap gene targets in embryos. First, as seen with the Hb expression boundary positions in wt embryos (Fig. 2C), targets at other boundary positions along the A-P axis (except the most anterior part of the embryo) should also exhibit a slanting effect, i.e., Δ*x*_Target _= *x*_TargetD _- *x*_TargetV _should be a positive value. Second, while targets with a boundary position closer to mid-embryo should have a smaller Δ*x*_Target _thus with a subdued slanting, those with a more anterior boundary position (expect near the anterior pole) should have a larger Δ*x*_Target _with an enhanced slanting. Third, the distribution of the lines connecting the boundary positions on the two sides of the embryo should exhibit a global similarity to that of Bcd profiles shown in Fig. 4B. Finally, as seen in Fig. 4A where Δ*x*_Bcd _is larger than Δ*c*_Bcd _except at the highest Bcd concentrations (which correspond to locations near the anterior pole of the embryo), Δ*x*_Target _should be larger than Δ*c*_Target_.

To directly evaluate these predictions, we measured *x*_Hb _in embryos that have either reduced (1×-*bcd*) or increased (3×-*bcd*) Bcd concentrations, thus shifting the *x*_Hb _position toward the anterior and posterior, respectively. We also analyzed the expression boundary of another direct Bcd target, Orthodenticle (Otd), in wt embryos. Similar to our analysis of Hb expression in wt embryos, we measured the expression boundary positions on both dorsal and ventral sides. Our results (Fig. 4C) show that, consistent with our predictions outlined above, Δ*x*_Target _is a positive value in all the cases tested. Furthermore, this value is highest for Otd, which has the most anterior expression boundary position among those tested, and lowest for *x*_Hb _in 3×-*bcd *embryos, which has the expression boundary position closest to the center of the embryo. The line distributions in Fig. 4D and Fig. 4B also exhibit a global similarity where data are available. Finally, as predicted and shown in Fig. 4C, Δ*c*_Target _(red) is smaller than Δ*x*_Target _(blue) in all cases tested (see additional file 1 for a theoretical discussion relevant to this issue). Together, these results show that the differences in Bcd gradient profiles on the dorsal and ventral sides of the embryos are responsible for the observed slanting of its direct gap gene targets.

### 3-D simulation of an embryo with asymmetric curvatures

We now return to the hypothesis that the Bcd gradient is formed in a process where contour distance from the anterior is measured, as suggested by our observed Bcd gradient properties in wt embryos at early nuclear cycle 14 (see also additional files 2, 3 and 4 for results in 1×-*bcd *and 3×-*bcd *embryos). Bcd gradient formation is a complex process, which takes place during a developmental period when the embryo itself is undergoing an exponential growth in terms of the nuclear number. There are also migrations of the nuclei to the cortex of the embryo at nuclear cycle 10, leading to the formation of the cortical (nuclear) layer where Bcd activates transcription of its downstream target genes [6,19]. How distance is measured and positional information encoded by the Bcd gradient in the embryo is governed by physical properties of both Bcd molecules and the embryo acting as the Bcd diffusion medium. We recently developed a biologically realistic 2-D model to simulate the dynamics of Bcd gradient formation in embryos [33]. This model was based on the equilibrium binding of Bcd molecules to low-affinity, non-specific DNA bindings sites throughout the *Drosophila *genome. Here we expand this 2-D model to a new 3-D model (see Methods for further details).

**Figure 5:**
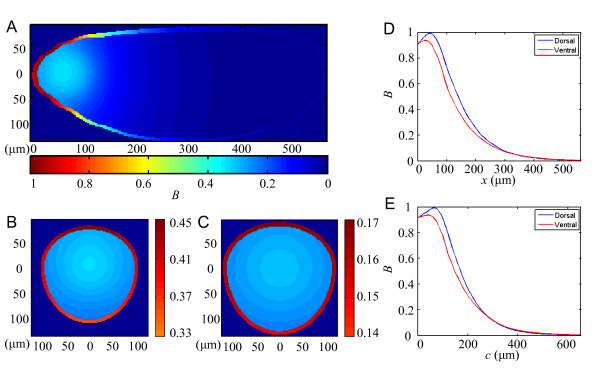
**Simulating the effect of embryo geometry on Bcd gradient profiles**. (A) A heat map of local total Bcd concentration on the midsagittal plane of a simulated embryo at nuclear cycle 14. The shape of the simulated embryo is based on the experimentally observed average wt embryos (Fig. 4B) with an asymmetric geometry on the dorsal and ventral sides (see Methods for details). Color bar represents normalized local total Bcd concentration. (B and C) Heat maps of local total Bcd concentration of the same simulated embryo on transverse planes at *x*_Otd_/*L *(panel B) and *x*_Hb_/*L *(panel C). Color bar represents normalized local total Bcd concentration only showing the range for the cortical layer relevant to our discussions. (D and E) Nuclear Bcd concentration profiles from the cortical layer of the dorsal (blue) and ventral (red) sides of the simulated embryo, measured as a function of either projected distance *x *from the anterior (panel D) or contour distance *c *(panel E). See text and Methods for further details.

To further study the effects of embryo geometry on Bcd gradient formation, and to specifically determine whether a curvature difference on the two sides of the embryo may be sufficient to explain the observed Bcd gradient properties, we performed simulations in our 3-D model. Fig. 5A shows a heat map of local total Bcd concentration on the midsagittal plane of a simulated embryo, at nuclear cycle 14, with asymmetric curvatures on its dorsal and ventral sides (see Methods for details). Fig. 5B and 5C show heat maps of local total Bcd concentration of the same simulated embryo on transverse planes at the Otd and Hb boundary positions. As seen experimentally [8,19,30,34], our simulated embryo shows that Bcd molecules are concentrated within the cortical layer where the nuclei are located. We analyzed the simulated nuclear Bcd concentration profiles within the cortical layer on both sides of the midsagittal plane in a manner identical to our analyses of the experimental data presented above. Fig. 5D and 5E show simulated nuclear Bcd concentration profiles as a function of projected distance *x *or contour distance *c*, respectively. Our simulated Bcd profiles recapitulate three experimentally observed properties regarding D-V differences (compared with experimental data shown in Fig. 2A and 2B). First, the value of maximal Bcd concentration on the ventral side is lower than that on the dorsal side (see also additional files 7 and 8 for additional simulating results). Second, the length constant on the ventral side is larger than on the dorsal side when measured as a function of either *x *or *c *(see below and additional file 8 for further details). Third, for a broad section of the embryo along the A-P axis, the simulated Bcd profiles on the two sides are more different as a function of *x *than as a function of *c*. In particular, similar to experimental results (Fig. 4A), Δ*c*_Bcd _at both the Hb and Otd boundary locations (2.6 *μ*m and 21.8 *μ*m, respectively) is smaller than their corresponding Δ*x*_Bcd _(13.7 *μ*m and 24.3 *μ*m, respectively; see additional file 8 for further details). These results demonstrate that a curvature difference between the two sides of an embryo is sufficient to cause distinct Bcd gradient properties similar to those observed experimentally. Importantly, our results show that the nuclear layer is not only where Bcd molecules are enriched but also a principal medium through which the Bcd gradient is formed in a syncytial blastoderm, providing an explanation for why it is more appropriate to describe Bcd gradient in contour distance *c *than in projected distance *x *(see below for further discussion on why the cortical layer may not be viewed as an isolated diffusion medium).

## Discussion

In this study we systematically analyze the Bcd and Hb behaviors on both the dorsal and ventral sides of the embryo. We reasoned that, since these two sides of the embryos have distinct geometric features (Fig. 1, see also additional file 1 for a theoretical discussion), a direct comparison between the Bcd and Hb profiles on these two sides should shed new light on how embryo geometry may affect both Bcd gradient formation and target gene activation during development. In our analyses we use two distinct methods to measure positions along the A-P axis: the projected distance *x *from the anterior pole and the contour distance *c *along the perimeters of the dorsal or ventral sides of the embryo. Our findings support a hypothesis that the Bcd gradient profile establishes and exerts the positional information by following the contour distance *c*. Our simulation studies (Fig. 5) show that a curvature difference as seen on the dorsal and ventral sides of the embryo is sufficient to generate Bcd gradient properties consistent with experimental data. Mechanistically, our findings underscore the importance of the nuclear layer on the cortex of a *Drosophila *syncytial blastoderm as a critical medium for Bcd gradient formation. However, as further discussed below, Bcd gradient formation cannot be viewed as a simple 1-D process in the cortical layer as an isolated diffusion medium, suggesting that an interaction (exchange of Bicoid molecules) between the cortical layer and the inner part of the embryo is important in proper Bcd gradient formation.

Our quantitative studies presented in this report show that, at early nuclear cycle 14, the dorsal and ventral sides of the embryo have a fundamentally similar input-output relationship between Bcd and Hb (see additional file 9 for experimentally measured parameter values). These results provide important insights into the well-documented splay of the segmentation gene expression stripes toward the poles on the ventral side [20-23]. Previous studies did not have a quantitative consideration of both the embryo geometry and morphogen profiles and, thus, were not able to explain how such a splay is achieved mechanistically. Our current work probes, in a quantitative way, the earliest decisions an embryo has to make along the A-P axis, namely, expression of gap genes in response to the Bcd gradient in the anterior. Our results show that the slanting of the Hb and Otd expression domains can be directly traced to the differences in Bcd gradient properties between the two sides of the embryo (Fig. 4). Our input-output analyses further support the notion that Bcd gradient properties are primarily responsible for target expression differences on the dorsal and ventral sides of the embryo. Additional support to this conclusion also comes from previous findings that a Bcd-responsive reporter containing only Bcd binding sites (thus responding to Bcd as the sole activator input), or even reporters of artificially-generated A-P activator gradients, also exhibit a slant of the expression boundary [34]. We suggest that the documented, extensive interactions between the A-P and D-V systems [26-28] may play important roles in maintaining or refining the expression of A-P genes that are either further downstream of the gene activation cascade or at times subsequent to early nuclear cycle 14, the stage of embryos analyzed in our work. We note that, unlike in the anterior (Fig. 4B), the iso-concentration contour lines of the Bcd gradient in the posterior part of the embryo do not exhibit any obvious ventral splay toward the posterior pole (data not shown). It is possible that the D-V system may play a particularly important role in A-P gene expression, in terms of ventral splay, in the posterior. This suggestion is consistent with recent quantitative findings that mutations in the D-V system preferentially reduce the splay of segmentation gene expression stripes in the posterior [25]. An alternative explanation is that posterior gradients such as Nanos may have D-V differences analogous to the Bcd gradient. We also note that recent studies suggest that the terminal system and other gap gene products can interact with Bcd to regulate target gene expression [35-40]. However, these interactions, which are along the A-P axis, do not affect our interpretations of the sources of D-V differences regarding the observed slanting of Bcd target expression patterns.

Our experimental and simulation results suggest that proper Bcd gradient formation may require an interaction between the cortical layer and the inner part of the embryo. In particular, we found that the Bcd gradient profiles on the two sides of the same embryo are not identical even when measured as a function of contour distance *c *(Fig. 2B, see also simulated results in Fig. 5E). Ideally, if Bcd diffusion within the cortical layer were a simple 1-D process, we would have expected Bcd profiles on both sides of the embryo to be identical when measured as a function of contour distance *c*. Our experimental results show that, compared with the dorsal side, the Bcd gradient on the ventral side has a lower *B*_max _(see Fig. 2A and 2B) and a larger length constant *λ *(calculated by fitting to *B_x _*= *B*_0_e^-*x*/*λ*^; Fig. 2A values measured in *x*: *λ*_D _= 99.2 ± 7.4 *μ*m, *λ*_V _= 112.6 ± 11.5 *μ*m; Fig. 2B values measured in *c*: *λ*_D _= 104.8 ± 6.9 *μ*m, *λ*_V _= 115.5 ± 9.0 *μ*m). These results indicate that simple 1-D diffusion models, even when incorporating the appropriate consideration that the cortical layer is a critical medium for Bcd diffusion, are inadequate to address the geometric issues in such a complex biological process. In contrast, our 3-D simulation with an asymmetric embryo revealed results that are strikingly similar to these experimental findings including a lower *B*_max _and longer *λ *on the ventral side than the dorsal side (Fig. 5D values measured in *x*: *λ*_D _= 92.3 *μ*m, *λ*_V _= 98.2 *μ*m; Fig. 5E values measured in *c*: *λ*_D _= 95.5 *μ*m, *λ*_V _= 105.8 *μ*m). These differences are also obtained in a 2-D simulation of an asymmetric embryo in which, similar to the simulated 3-D embryo, Bcd molecules can diffuse within the inner part of the embryo and are allowed to exchange between the inner part and the cortical layer (not shown). However, in a 3-D simulation where the inner part of the embryo is impermeable to Bcd molecules (thus no exchange between the inner part and the cortical layer), the simulated *λ*_V _is not larger than *λ*_D _(not shown). We note that, although our 3-D simulation can replicate our experimentally observed Bcd gradient properties, it is possible that other physical features of the embryo may exert additional effects on Bcd gradient formation. For example, it has been shown that the ventral side of wt embryos has a lower nuclear density than the dorsal side [25,41]. It is possible that the nuclear density difference may further affect Bcd gradient formation in ways (e.g., Bcd molecule diffusion) that are beyond those caused by the geometric differences, an issue that will require future investigations. Furthermore, nuclei on the cortex of the embryo at nuclear cycle 14 make systematic movements referred to as the nuclear flow [25] but such a flow was shown to have a relatively minor effect on the anterior Hb expression pattern. We emphasize that, since our current 3-D model can fully recapitulate our experimentally observed properties regarding the differences between the dorsal and ventral sides of the embryo, it likely has incorporated the key physical quantities most relevant to the system behavior.

## Conclusions

The experimental and theoretical studies described in this report represent a first, systematic investigation of the effects of embryo geometry on the positional information encoded by the Bcd gradient. Our results suggest that the ventral splay of segmentation genes along the A-P axis may be directly traced to the distinct properties of the Bcd gradient (and its gap target gene responses) on the dorsal and ventral sides of the embryo. Our findings also suggest that, while the cortical layer is critical for Bcd gradient formation, Bcd diffusion likely requires an interaction between the cortical layer and the inner part of the embryo. These results demonstrate that modeling a morphogen gradient requires a realistic consideration of the physical properties, including the geometry, of the relevant biological system.

## Methods

### Immunostaining and microscopy

In our experiments, embryos were collected, fixed and stained with antibodies as described previously [8]. 1×-*bcd *embryos were from *bcd^E1^*/+ females and 3×-*bcd *embryos were from *CyO, bcd*/+ females (gift of Gary Struhl). For Bcd-Hb-DAPI triple staining, we used rabbit anti-Bcd [42] and guinea pig anti-Hb [43] as primary antibodies, and Alexa-594-conjugated goat anti-rabbit and Alexa-488-conjugated goat anti-guinea pig antibodies (Molecular Probes) as secondary antibodies. DAPI (Sigma) was used to stain the nuclei to facilitate imaging and data processing. For Otd-Hb-DAPI triple staining, we used guinea pig anti-Otd [44] and rat anti-Hb [43] as primary antibodies, and Alexa-488-conjugated goat anti-guinea pig and Alexa-555-conjugated goat anti-rat antibodies (Molecular Probes) as secondary antibodies. High-resolution images (1388 × 1040 8 bits/pixel) were captured on Zeiss Imager Z1 ApoTome microscope with a Zeiss Plan Apochromat 20× objective in linear settings (i.e, without any normalization during the imaging stage). Throughout this work, we use raw Bcd intensities that were captured within a linear range (i.e, without any saturation), and there were no further adjustments, except background subtraction when stated (see additional file 5 for raw Bcd intensity data). To ensure that background was measured under identical experimental conditions, we mixed experimental embryos with those from *bcd^E1 ^*females (thus lacking Bcd). In our study, the mean Bcd intensities of *bcd^E1 ^*embryos were simply subtracted as background from the intensity data of the experimental embryos. All stained embryos were mounted under cover slips with bridges to avoid geometric distortions, and images were taken on the midsagittal plane. The estimated noise in *L *measurement caused by focal plane selection is <0.1% [8], which is negligible compared with the measured differences between *L *and *C*_D _(~5%) or between *C*_D _and *C*_V _(~13%) in our current study.

### Measurements of distance and fluorescence intensities

All results presented in this study were from embryos at early nuclear cycle 14 with an approximated time window of 6-24 minutes after mitosis, which were selected based on nuclear number, size and shape, and the posterior Hb level as described previously [8]. DAPI images were used to locate the embryo and its cortical layer and to define the A-P poles. Intensity scanning was performed in an automated way using MatLab as follows. After the A-P axis was established, a circular window of the size (~25 *μ*m^2^) smaller than that of a nucleus was allowed to slide along the entire nuclear layer, on both the dorsal and ventral sides of the embryo images. The center of the scanning window was kept ~6 *μ*m away from the perimeter of the embryo, with a pixel-by-pixel sliding along the A-P axis projection (Fig. 1B). At each position, the *x*-*y *coordinates were record, and the raw fluorescence intensities at different channels (except DAPI channel) were extracted from the images. For each embryo, the contour distance *c *from the anterior is computed as:(2)

where *j *is the index of the scanning windows. Raw Bcd intensities were obtained by averaging the pixel intensities within the scanning windows, without further adjustments except background subtraction when necessary (Fig. 3B and 3C). The dorsal and ventral sides have similar background Bcd intensities as measured from *bcd^E1 ^*embryos (2.19 ± 0.20 and 2.13 ± 0.22 for the dorsal and ventral sides, respectively; *p *= 0.27). In our analysis, the measured fluorescence intensities for both Hb and Otd were normalized by setting the mean of the highest values to 1 and the mean at non-expression regions to 0. See additional file 6 for Hb intensity profiles. Normalization of Hb intensities is required for our analysis, as well as in all published work in the field. This is due to the high variability of absolute Hb intensities among different embryos. However, since Hb expression has a clear boundary, unlike the Bcd gradient, such normalization is expected to cause problems that are relatively minor compared to normalization of the Bcd intensities. All calculations were performed using MatLab as described previously [8].

### Plotting iso-intensity lines of Bcd onto an average embryo frame

We first compute the average shape of embryos by plotting the mean of normalized D-V positions along the perimeters of the embryos against the corresponding *x*/*L*. To compute the iso-intensity contour lines for Bcd, we choose a series of intensity values within the range of measured raw Bcd intensities as thresholds (*t_k_*) to identify positions (*x_k_*, *y_k_*) where *B*_D _= *B*_V _= *t_k _*by interpolation. Then we plot these positions onto the average embryo frame and connect the positions on the dorsal and ventral sides to visualize the distribution of iso-intensity contour lines (Fig. 4B). The iso-intensity lines are drawn for the anterior half of the embryo, where Bcd function is primarily relevant. In order to spread the iso-intensity lines for viewing quality, we use different Bcd intensity value intervals (*t*_*k *+ 1 _- *t_k_*) for 1×-*bcd*, wt and 3×-*bcd *embryos (0.5, 1 and 1.5, respectively), whose average frames are calculated separately (N = 24, 28 and 29, respectively). For visual comparison, we also plot lines connecting the target boundary positions on two sides of the embryos using normalized average frames of 1×-*bcd*, wt and 3×-*bcd *embryos in the same presentation (Fig. 4D).

### 3-D simulation

We use a 3-D simulation model, which is based on our recently developed 2-D model [33], to analyze the effects of both embryo geometry and cortex-yolk interaction on Bcd gradient formation. Briefly, our model is based on the equilibrium binding of Bcd molecules to non-specific, low affinity DNA binding sites throughout the *Drosophila *genome. In our model, Bcd molecules are produced at a constant rate within a *bcd *mRNA sphere located in the anterior. Unbound Bcd molecules at one location are allowed to diffuse to and from its six immediate neighboring locations thus truly in 3-D. Our model also considers the doubling of the nuclear number after each nuclear cycle and their relative locations (i.e., they are evenly distributed within the embryo prior to nuclear cycle 10, but migrate to the cortex to form a cortical layer at the onset of nuclear cycle 10). In our current work, an embryo was selected to resemble the average shape of a wt embryo with its dorsal and ventral curvatures on the midsagittal plane directly extracted from experimental measurements (Fig. 4B). This simulated embryo, which is shown in Fig. 5, is 560 *μ*m in length and has a lateral diameter of 220 *μ*m. We also simulated another embryo using an asymmetric approximation for its shape [45,46] and obtained comparable results (see additional file 7 and the legend for further details).

In our model, the anterior pole of the simulated embryo has a coordinate of (0, 0, 0), and the *bcd *mRNA sphere from which Bcd is produced has a radius of 45 *μ*m and a center coordinate of (75 *μ*m, 0, 0). We tested our model by making reasonable changes in the shape or size of the mRNA distribution and obtained consistent Bcd protein gradient properties (not shown). The parameter value for the aggregate Bcd synthesis rate *J *for the entire embryo in our current model does not affect the experimentally observed three properties discussed in the main text (not shown), and we chose *J *= 1000 molecules/s so that at cycle 14, the maximum nuclear Bcd concentration on the dorsal side is 37.1 molecules/μm^3^, a value consistent with experimental estimates [6]. The cortical layer has a depth of 10 *μ*m in our current model and was kept unchanged between nuclear cycles 10 to 14; our simulations using thicker cortical layer similarly yielded the three experimentally observed properties (not shown). The simulated developmental time is based on experimental observations [18,19], and the simulated results shown in Fig. 5 and additional file 7 are at 14 minutes into nuclear cycle 14. Other parameters used in our model are: the diffusion constant for free Bcd molecules, *D *= 2 *μ*m^2^s^-1^; the spatially-uniform degradation rate of Bcd, *ω *= 0.00005 s^-1^, and the ratio of DNA-bound to free local Bcd concentrations at nuclear cycle 10, *B*_bound_/*B*_free _= 0.15. As further detailed in [33], these parameter values were chosen through a systematic sampling such that the simulated Bcd profiles exhibit properties consistent with those observed experimentally, including proper *λ *values of the simulated Bcd profiles [6,8], an enrichment of the Bcd molecules in the cortical layer [19], and stable nuclear Bcd concentrations at nuclear cycle 10-14 [19]. These chosen values are biologically reasonable and, as shown in [33], are adjustable within our identified parameter space. In our simulations we tested grid size of 1^3^, 2^3 ^and 5^3 ^μm^3 ^and Δ*t *= 0.1, 0.2, and 0.5 s, and obtained consistent results of Bcd gradient behaviors, indicating that our solutions are numerically stable. Results in Fig. 5 and additional file 7 were obtained with a grid size of 2^3 ^μm^3 ^and Δ*t *= 0.2 s. We also conducted simulations to evaluate the effects of other parameters on simulated Bcd gradient properties. As shown in additional file 8 (see legend for details), movements of the *bcd *mRNA sphere's center location along the A-P or D-V axis (*Rx *and *Ry*, respectively) or the *B*_bound_/*B*_free _ratio at nuclear cycle 10, while affecting the specific values of the simulated profiles as expected, yielded results that remain generally consistent with the three experimentally observed properties. Together, these results show that our simulated Bcd properties that are in agreement with those observed experimentally are robust to reasonable variations (e.g., in *bcd *mRNA shape, size and location) that may naturally exist among real embryos.

## Authors' contributions

FH and JM conceived and designed the study. FH, YW and DC performed experiments. FH analyzed data. FH, LJL, RJ, and JM interpreted results. FH and JD developed the theoretical model and conducted simulations. FH generated all figures. FH and JM wrote the paper. All approved the paper.

## Supplementary Material

Additional file 1 **Theoretical 2-D consideration of geometry and positional information**. Click here for file

Additional file 2 **Figure S1: Analysis of D-V differences for Bcd and Hb profiles in 1×-*bcd *embryos**. (A and B) Average raw Bcd intensity profiles on the dorsal (blue) and ventral (red) sides of 24 1×-*bcd *embryos when measured as a function of projected distance *x *from the anterior (A) or contour distance *c *(B). In each panel, the inset shows a magnified view of the region surrounding the Hb expression boundary positions (solid arrowheads). Listed below are *p *values from Student's t-tests at the indicated locations in the insets: 10^-5^, 10^-5^, 0.0003, 0.002, and 0.02 for panel A, and 0.004, 0.0004, 0.002, 0.01 and 0.05 for panel B. (C and D) Average normalized Hb intensity profiles on the dorsal (blue) and ventral (red) sides of the same 1×-*bcd *embryos when measured as a function of projected distance *x *from the anterior (C) or contour distance *c *(D). See Additional file 9-Table S1 for measured values. (E-H) Bcd-Hb input/output relationship of 1×-*bcd *embryos analyzed in a scatter plot (E), average input-output relationship (F), profiles of Hb expression noise in response to Bcd concentration (G) and Bcd intensity noise (H). These analyses, together with those obtained in wt embryos (Fig. 3) and 3×-*bcd *embryos (Fig. S2), reveal a fundamentally similar Bcd-Hb relationship on both sides of the embryos. See Additional file 9- Table S1 for measured values. Click here for file

Additional file 3 **Figure S2: Analysis of D-V differences for Bcd and Hb profiles in 3×-*bcd *embryos**. (A and B) Same as panels A and B of Fig. S1, except the data are from 29 3×-*bcd *embryos. Listed below are *p *values from Student's t-tests at the indicated locations in the insets: 0.01, 0.02, 0.01, 0.02, and 0.13 for panel A, and 0.15, 0.39, 0.81, 0.36 and 0.71 for panel B. (C-H) Same as panels C-H of Fig. S1, except the data are from 3×-*bcd *embryos. See Additional 9- Table S1 for measured values. Click here for file

Additional file 4 **Figure S3: Analysis of distance measurements in 1× and 3×-*bcd *embryos**. (A and C) Profiles of distance differences between the dorsal and ventral sides of the 1×-*bcd *(A) and 3×-*bcd *(C) embryos when measured as either projected distance from the anterior (blue, left scale) or contour distance (red, right scale). (B and D) Iso-concentration contour lines for Bcd plotted on the average frames for 1×-*bcd *(B) or 3×-*bcd *(D) embryos, respectively. The intensity increments between neighboring contour lines (red) are 0.5 (B) and 1.5 (D), respectively. See main text and Fig. 4A legend for further details. Click here for file

Additional file 5 **Figure S4: Raw Bcd intensity data**. (A and B) Raw Bcd intensity profiles on the dorsal (blue) and ventral (red) sides of 24 1×-*bcd *embryos when measured as a function of projected distance *x *from the anterior (A) or contour distance *c *(B). (C and D) Same as panels A and B, except the data are from 28 wt embryos. (E and F) Same as panels A and B, except the data are from 29 3×-*bcd *embryos. Click here for file

Additional file 6 **Figure S5: Normalized Hb intensity data**. (A and B) Normalized Hb intensity profiles on the dorsal (blue) and ventral (red) sides of 24 1×-*bcd *embryos when measured as a function of projected distance *x *from the anterior (A) or contour distance *c *(B). (C and D) Same as panels A and B, except the data are from 28 wt embryos. (E and F) Same as panels A and B, except the data are from 29 3×-*bcd *embryos. Click here for file

Additional file 7 **Figure S6: Simulating an embryo that has an asymmetric geometry**. (A) A heat map of local total Bcd concentration on the midsagittal plane of a simulated embryo, which is approximated by two semi-ellipsoids joined on the coronal plane, with a height of 92 *μ*m and 128 *μ*m for the dorsal and ventral sides, respectively, and a length of 560 *μ*m. For consistency, all other parameters here are set the same as those used to generate the results shown in Fig. 5. Color bar represents normalized local total Bcd concentration. (B and C) Heat maps of local total Bcd concentration of the same simulated embryo on transverse planes at *x*_Otd_/*L *(panel B) and *x*_Hb_/*L *(panel C). Color bar represents normalized local total Bcd concentration only showing the range for the cortical layer. (D and E) Nuclear Bcd concentration profiles from the cortical layer of the dorsal (blue) and ventral (red) sides of the simulated embryo, measured as a function of either projected distance *x *from the anterior (panel D) or contour distance *c *(panel E). Click here for file

Additional file 8 **Figure S7: Testing the effects of *bcd *mRNA location and DNA binding on Bcd gradient properties**. In these simulations, the center location of the *bcd *mRNA sphere was systematically moved either along the A-P axis (panels A-C; *Rx *denotes distance from anterior pole) or along the D-V axis (panels D-F; *Ry *denotes distance from the A-P axis). We also systematically changed the ratio of *B*_bound_/*B*_free _at nuclear cycle 10 (panels G-I). Here, the shape of the simulated embryo is the same as in Fig. 5; for reference, the parameter values used in the simulations shown in Fig. 5 and Fig. S6 are marked with arrowheads. The resulting Bcd gradient profiles on the dorsal and ventral sides were then evaluated for their *B*_max _values (A, D and G), *λ *values expressed as either *c *or *x *(B, E and H), and the values of Δ*x*_Bcd _and Δ*c*_Bcd _at target boundary positions of both Hb and Otd (C, F and I). As shown in individual panels, these simulated results remain consistent with the three experimentally observed properties: 1) *B*_max _is higher on the dorsal side than on the ventral side (A, D and G), 2) *λ *measured as either *x *or *c *is larger on the ventral side than on the dorsal side (B, E and H; compare dashed lines with solid lines), and 3) Δ*x*_Bcd _at target boundary locations is larger than their corresponding Δ*c*_Bcd _(C, F and I; compare blue lines with red lines). As further discussed in the theoretical consideration above, the value of Δ*x*_Bcd _- Δ*c*_Bcd _in the middle section of the embryo reflects the geometric properties of the embryo. This value is 11.1 *μ*m at *x*_Hb_/*L *in our simulation results shown in this figure and, as expected, is insensitive to *bcd *mRNA location or the *B*_bound_/*B*_free _ratio. It is also fully consistent with both experimental (10.7 *μ*m as shown in Fig. 4A) and theoretical values (11.7 *μ*m as shown in Fig. 1C). Click here for file

Additional file 9 **Table S1: Experimentally determined parameter values for the Bcd-Hb relationship**. Click here for file
