# Supplementary material for: Distance measurements via the morphogen gradient of Bicoid in Drosophila embryos
Source: BMC Dev Biol. 2010 Aug 2;10:80. doi: 10.1186/1471-213X-10-80 (PMC2919471; doi:10.1186/1471-213X-10-80)
Supplement: Additional file 1 — Theoretical 2-D consideration of geometry and positional information. [file 1471-213X-10-80-S1.PDF]

Additional file 1: Theoretical 2-D consideration of geometry and positional information.

Here we present a theoretical analysis of the relationship between embryo geometry and positional information encoded by the Bcd gradient. This analysis is based on a 2-D consideration of the embryo on the midsagittal plane, where our experimental data are available. Since the curvatures for the two sides of an embryo on this plane are different as shown experimentally (Fig. 1B), we use functions $f_1(x)$ and $f_2(x)$ to denote the conversions between the contour distance *c* and projected distance *x* for the dorsal and ventral sides, respectively. To begin our analysis, we first find locations on the dorsal and ventral sides of the embryo where *B* values are identical. These locations, as a function of *B*, are expressed as either contour distances from the anterior pole, $c_D(B)$ and $c_V(B)$ for the dorsal and ventral sides, respectively; or projected distances from the same anterior pole, $x_D(B)$ and $x_V(B)$ for the dorsal and ventral sides, respectively. Here we consider only the regions of the embryo where the Bcd concentration decreases between any intervals from an anterior location to a posterior location to ensure that the conversions from Bcd concentration to position have unique solutions. We define $\Delta c(B) = c_D(B) - c_V(B)$ and $\Delta x(B) = x_D(B) - x_V(B)$. Note that $\Delta c(B)$, which is a function of *B* here, should not be confused with Δc_{embryo} (of Fig. 1C), which is a function of *x*. Here, we introduce a term, equivalent to Δc_{embryo}, to define the geometric differences between the dorsal and ventral sides of the embryo: $g(x) = f_1(x) - f_2(x)$ (see Fig. 1C for experimentally determined *g(x)* profile in wt embryos). By converting projected distances into contour distances, we have:

$$\begin{aligned}\Delta c(B) &= c_D - c_V \\ &= f_1(x_D) - f_2(x_V) \\ &= f_1(x_D) - f_2(x_D) + f_2(x_D) - f_2(x_V) \\ &= g(x_D) + f_2(x_D) - f_2(x_V)\end{aligned} \qquad \text{Eq. S1}$$

According to the mean value theorem, there exists at least one point ξ in the interval (x_D, x_V) such that

$$f_2(x_D) - f_2(x_V) = f_2'(\xi)(x_D - x_V) = f_2'(\xi)\Delta x(B)\,. \qquad \text{Eq. S2}$$

Combining Eqs. S1 and S2, we get

$$\Delta c(B) = g(x_D) + f_2'(\xi)\Delta x(B)\ . \qquad \text{Eq. S3}$$

Eq. S3 can also be expressed in an alternative way as follows:

$$\Delta c(B) = g(x_V) + f_1'(\zeta)\Delta x(B)\,, \qquad \text{Eq. S4}$$

where ζ is a point in the interval (x_D, x_V) according to the mean value theorem.

As further discussed in the main text, $\Delta x(B)$ effectively represents a tilt or slant of the positional information encoded by the Bcd gradient (and consequently its target responses) on the two sides of the embryo. $\Delta x(B)$ can be written as:

$$\Delta x(B) = \frac{\Delta c(B) - g(x_D)}{f_2'(\xi)}\,. \qquad \text{Eq. S5}$$

Here, we focus on the anterior part of the embryo, where the Bcd gradient is primarily relevant to patterning and target expression boundary positions are experimentally

measured, to evaluate the relative contributions of $\Delta c(B)$, $g(x_D)$ and $f_2^{'}(\xi)$ to the slanting of positional information encoded by the Bcd gradient. We use our Bcd intensity data obtained from wt embryos in our analysis. Since Bcd intensity profiles in embryos from females that have different *bcd* gene doses exhibit a linear relationship along the entire A-P length [1, 2], we also analyze the Bcd system behavior at Hb expression boundary positions obtained from 1×-*bcd* and 3×-*bcd* embryos. As shown in additional file 4B and 4D, the profiles of iso-concentration lines in 1×-*bcd* and 3×-*bcd* embryos are virtually the same as that of wt embryos (Fig. 4B) when intensity intervals between the lines are adjusted for the *bcd* gene dose in the females. In our analysis, we simply use Hb boundary locations in 1×-*bcd* and 3×-*bcd* embryos as mere positions to evaluate the wt Bcd gradient behaviors at these locations without implying that we could use Bcd concentration values from wt embryos at such locations to describe the input-output relationship between Bcd and Hb in 1×-*bcd* and 3×-*bcd* embryos.

For Hb in 1×-*bcd*, wt and 3×-*bcd* embryos, the Hb expression boundary positions are located within the broad "middle" section of the embryos discussed in the main text, $0.36 < x/L < 0.66$ (Hb mean boundary positions $x_D/L =$ 0.37, 0.44 and 0.49, respectively). Within this "middle" section of the embryo, the curvature on either dorsal or ventral side of the embryo is close to zero and, therefore, we make an approximation $f_2^{'}(\xi) \approx 1$. From Fig. 1C and Fig. 4A, we also know that, respectively, $g(x) < 0$ and $\Delta c(B) > 0$. Under these conditions, we have $\Delta x(B) \approx \Delta c(B) + |g(x_D)|$, suggesting that, within the "middle" section of the embryo, both $\Delta c(B)$ and $g(x_D)$ contribute to the 2-D

slanting pattern of the positional information of the Bcd gradient (Fig. 4B) and, consequently, the expression boundaries of its targets (Fig. 4D). Furthermore, within this broad "middle" section of $0.36 < x/L < 0.66$, we find that $\Delta x(B) - \Delta c(B) \approx |g(x_D)|$ remains approximately as a constant: $|g(x)| = 11.7 \pm 0.5$ μm as measured experimentally (see Fig. 1C and the main text) and results in our 3-D model (see also additional file 8 and its legend). These results suggest that, within this region of the embryo, the profile of $\Delta x(B)$ takes the shape of the profile of $\Delta c(B)$, which is fully consistent with our experimental data shown in Fig. 4A and C.

Otd in wt embryos has an expression boundary position that is most anterior among those tested in this work. Furthermore, it is located outside the "middle" section of the embryo as defined above (mean boundary positions for Otd: $x_D / L = 0.28$ and $x_V / L = 0.22$). From our calculations based on experimental data, we find that, at positions where Bcd concentrations reach a threshold for activating Otd expression, $\Delta c(B) = 30.3$ μm, $g(x_D) = -7.8$ μm and $1.13 < f_2(\xi) < 1.16$. Applying these values to Eq. S5, we get $32.8\,\mu\text{m} \le \Delta x(B) \le 33.7$ μm, which agrees fully with our experimentally determined value of $\Delta x(B) = 33.5$ μm.

In conclusion, our 2-D theoretical consideration of the relationship between embryo geometry and the positional information encoded by the Bcd gradient is consistent with our experimental findings. We note that, while both $g(x_D)$ and $f_2^{'}(\xi)$

represent pure geometric features of the embryo, $\Delta c(B)$ represents the difference in the Bcd gradient itself between the dorsal and ventral sides of the embryo. It is important to stress that $\Delta c(B)$ is also likely a result of the geometric differences between the two sides of the embryo (but it must be manifested through the gradient formation process) because our 3-D simulation results show that a curvature difference is sufficient to cause Bcd gradient profiles to be different, when measured as a function of *c*, between the two sides of the embryo (Fig. 5E, and see main text for further details). These considerations underscore the importance of embryo geometry in morphogen gradient formation and pattern formation.

Supplementary References

1. Gregor T, Tank DW, Wieschaus EF, Bialek W: **Probing the limits to positional information**. *Cell* 2007, **130**(1):153-164.
2. He F, Wen Y, Deng J, Lin X, Lu J, Jiao R, Ma J: **Probing intrinsic properties of a robust morphogen gradient in Drosophila**. *Dev Cell* 2008, **15**:558-567.
