# Supplementary material for: Distance measurements via the morphogen gradient of Bicoid in Drosophila embryos
Source: BMC Dev Biol. 2010 Aug 2;10:80. doi: 10.1186/1471-213X-10-80 (PMC2919471; doi:10.1186/1471-213X-10-80)
Supplement: Additional file 2 — Figure S1: Analysis of D-V differences for Bcd and Hb profiles in 1×-bcd embryos. (A and B) Average raw Bcd intensity profiles on the dorsal (blue) and ventral (red) sides of 24 1×-bcd embryos when measured as a function of projected distance x from the anterior (A) or contour distance c (B). In each panel, the inset shows a magnified view of the region surrounding the Hb expression boundary positions (solid arrowheads). Listed below are p values from Student's t-tests at the indicated locations in the insets: 10-5, 10-5, 0.0003, 0.002, and 0.02 for panel A, and 0.004, 0.0004, 0.002, 0.01 and 0.05 for panel B. (C and D) Average normalized Hb intensity profiles on the dorsal (blue) and ventral (red) sides of the same 1×-bcd embryos when measured as a function of projected distance x from the anterior (C) or contour distance c (D). See Additional file 9-Table S1 for measured values. (E-H) Bcd-Hb input/output relationship of 1×-bcd embryos analyzed in a scatter plot (E), average input-output relationship (F), profiles of Hb expression noise in response to Bcd concentration (G) and Bcd intensity noise (H). These analyses, together with those obtained in wt embryos (Fig. 3) and 3×-bcd embryos (Fig. S2), reveal a fundamentally similar Bcd-Hb relationship on both sides of the embryos. See Additional file 9- Table S1 for measured values. [file 1471-213X-10-80-S2.PDF]

Additional File 2

Figure S1. Analysis of D-V differences for Bcd and Hb profiles in 1×-*bcd* embryos

(A and B) Average raw Bcd intensity profiles on the dorsal (blue) and ventral (red) sides of 24 1×-*bcd* embryos when measured as a function of projected distance x from the anterior (A) or contour distance c (B). In each panel, the inset shows a magnified view of the region surrounding the Hb expression boundary positions (solid arrowheads). Listed below are p values from Student's t-tests at the indicated locations in the insets: 10^{-5}, 10^{-5}, 0.0003, 0.002, and 0.02 for panel A, and 0.004, 0.0004, 0.002, 0.01 and 0.05 for panel B.

(C and D) Average normalized Hb intensity profiles on the dorsal (blue) and ventral (red) sides of the same 1×-*bcd* embryos when measured as a function of projected distance x from the anterior (C) or contour distance c (D). See Table S1 for measured values.

(E-H) Bcd-Hb input/output relationship of 1×-*bcd* embryos analyzed in a scatter plot (E), average input-output relationship (F), profiles of Hb expression noise in response to Bcd concentration (G) and Bcd intensity noise (H). These analyses, together with those obtained in wt embryos (Fig. 3) and 3×-*bcd* embryos (Fig. S2), reveal a fundamentally similar Bcd-Hb relationship on both sides of the embryos. See Table S1 for measured values.

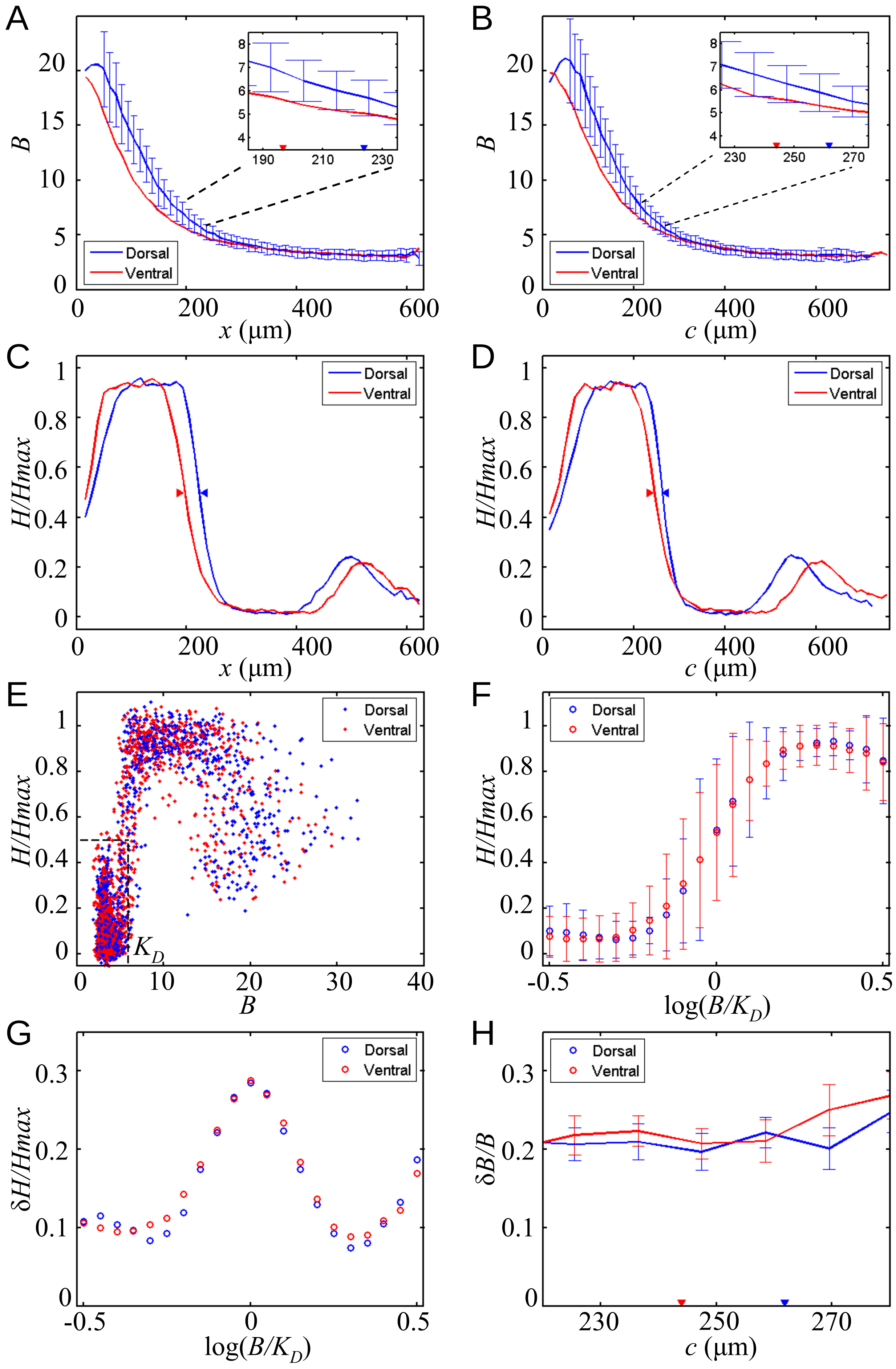

A
B
C
D
E
F
G
H
Dorsal
Ventral
B
x (μm)
c (μm)
H/Hmax
K_D
log(B/K_D)
δH/Hmax
δB/B
