# Supplementary material for: Distance measurements via the morphogen gradient of Bicoid in Drosophila embryos
Source: BMC Dev Biol. 2010 Aug 2;10:80. doi: 10.1186/1471-213X-10-80 (PMC2919471; doi:10.1186/1471-213X-10-80)
Supplement: Additional file 3 — Figure S2: Analysis of D-V differences for Bcd and Hb profiles in 3×-bcd embryos. (A and B) Same as panels A and B of Fig. S1, except the data are from 29 3×-bcd embryos. Listed below are p values from Student's t-tests at the indicated locations in the insets: 0.01, 0.02, 0.01, 0.02, and 0.13 for panel A, and 0.15, 0.39, 0.81, 0.36 and 0.71 for panel B. (C-H) Same as panels C-H of Fig. S1, except the data are from 3×-bcd embryos. See Additional 9- Table S1 for measured values. [file 1471-213X-10-80-S3.PDF]

Additional File 3

Figure S2. Analysis of D-V differences for Bcd and Hb profiles in 3×-*bcd* embryos

(A and B) Same as panels A and B of Fig. S1, except the data are from 29 3×-*bcd* embryos. Listed below are *p* values from Student's t-tests at the indicated locations in the insets: 0.01, 0.02, 0.01, 0.02, and 0.13 for panel A, and 0.15, 0.39, 0.81, 0.36 and 0.71 for panel B.

(C-H) Same as panels C-H of Fig. S1, except the data are from 3×-*bcd* embryos. See Table S1 for measured values.

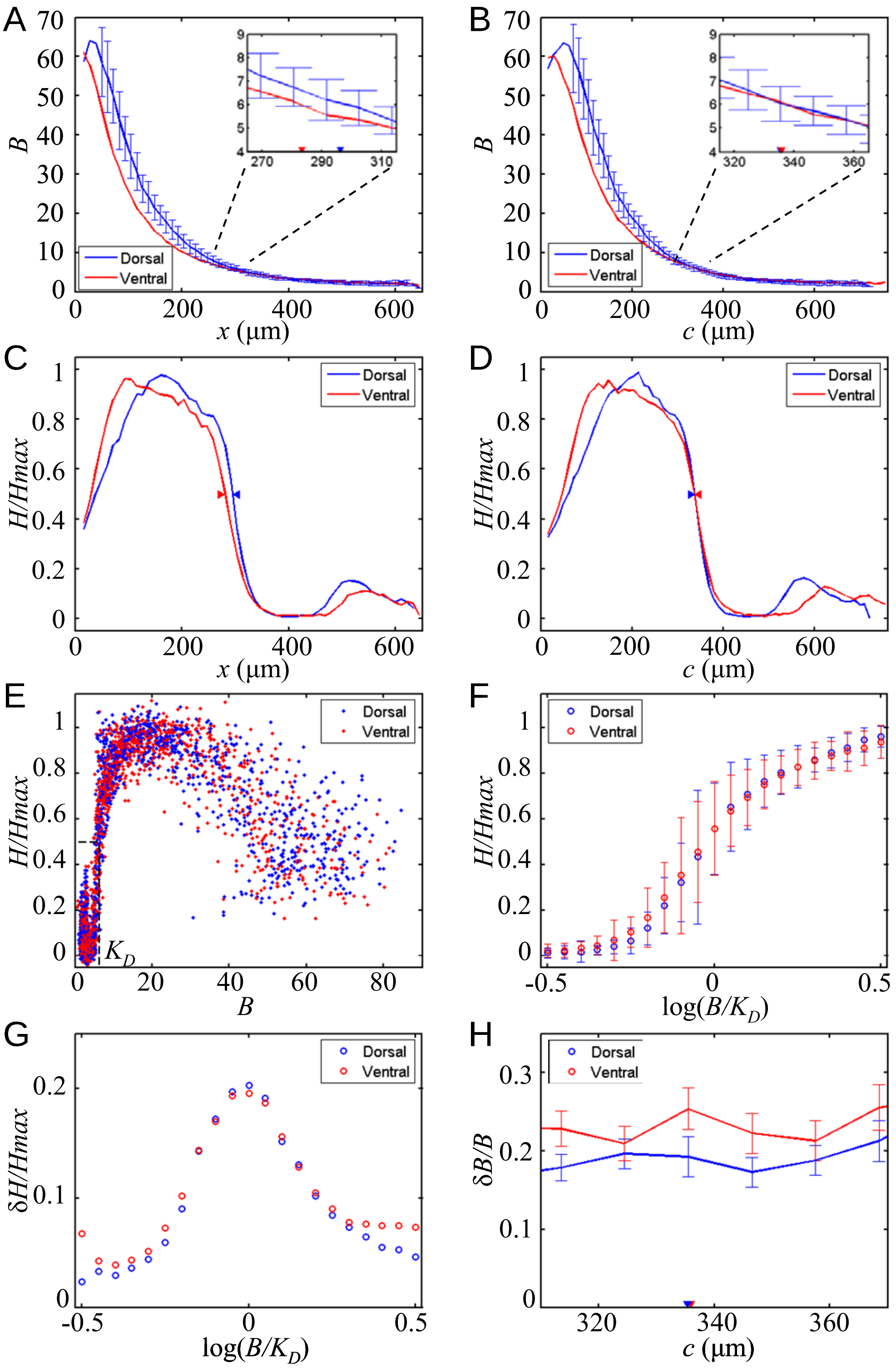

A
B
x (µm)
Dorsal
Ventral
B
c (µm)
C
H/Hmax
D
E
K_D
F
log(B/K_D)
G
δH/Hmax
H
δB/B
