# Supplementary material for: Distance measurements via the morphogen gradient of Bicoid in Drosophila embryos
Source: BMC Dev Biol. 2010 Aug 2;10:80. doi: 10.1186/1471-213X-10-80 (PMC2919471; doi:10.1186/1471-213X-10-80)

Additional File 4

Figure S3. Analysis of distance measurements in 1× and 3×-*bcd* embryos

(A and C) Profiles of distance differences between the dorsal and ventral sides of the 1×-*bcd* (A) and 3×-*bcd* (C) embryos when measured as either projected distance from the anterior (blue, left scale) or contour distance (red, right scale).

(B and D) Iso-concentration contour lines for Bcd plotted on the average frames for 1×-*bcd* (B) or 3×-*bcd* (D) embryos, respectively. The intensity increments between neighboring contour lines (red) are 0.5 (B) and 1.5 (D), respectively. See main text and Fig. 4A legend for further details.

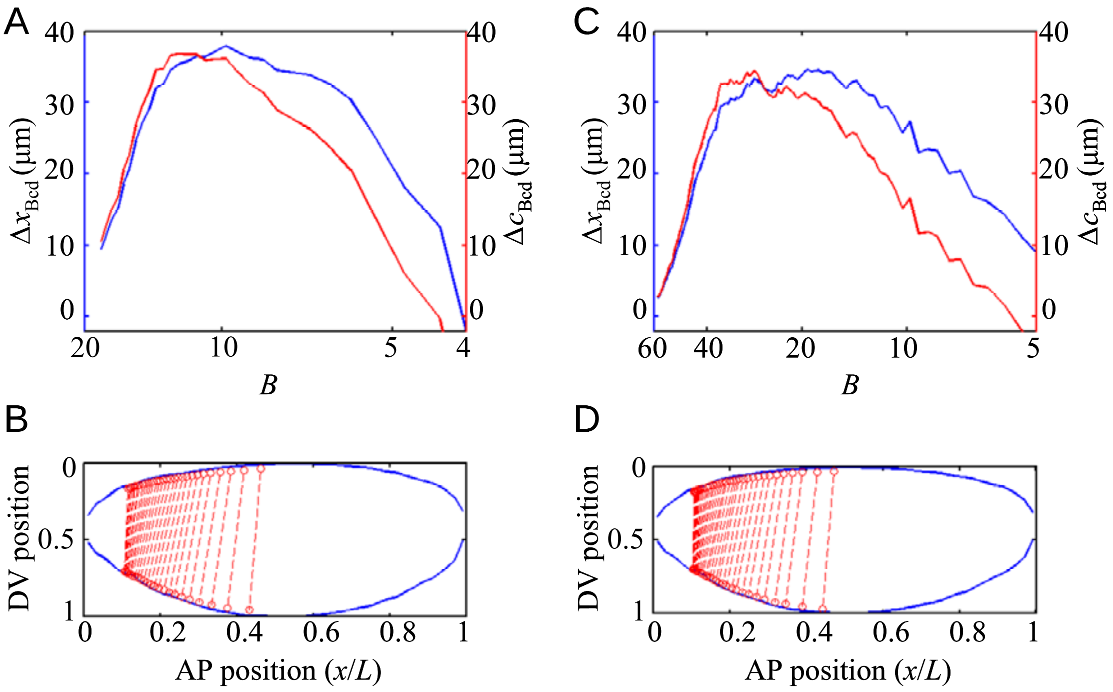

Supplement: Additional file 4 — Figure S3: Analysis of distance measurements in 1× and 3×-bcd embryos. (A and C) Profiles of distance differences between the dorsal and ventral sides of the 1×-bcd (A) and 3×-bcd (C) embryos when measured as either projected distance from the anterior (blue, left scale) or contour distance (red, right scale). (B and D) Iso-concentration contour lines for Bcd plotted on the average frames for 1×-bcd (B) or 3×-bcd (D) embryos, respectively. The intensity increments between neighboring contour lines (red) are 0.5 (B) and 1.5 (D), respectively. See main text and Fig. 4A legend for further details. [file 1471-213X-10-80-S4.PDF]
