# Supplementary material for: Distance measurements via the morphogen gradient of Bicoid in Drosophila embryos
Source: BMC Dev Biol. 2010 Aug 2;10:80. doi: 10.1186/1471-213X-10-80 (PMC2919471; doi:10.1186/1471-213X-10-80)

Additional File 5

Figure S4. Raw Bcd intensity data.

(A and B) Raw Bcd intensity profiles on the dorsal (blue) and ventral (red) sides of 24 1×-*bcd* embryos when measured as a function of projected distance x from the anterior (A) or contour distance c (B). (C and D) Same as panels A and B, except the data are from 28 wt embryos. (E and F) Same as panels A and B, except the data are from 29 3×-*bcd* embryos.

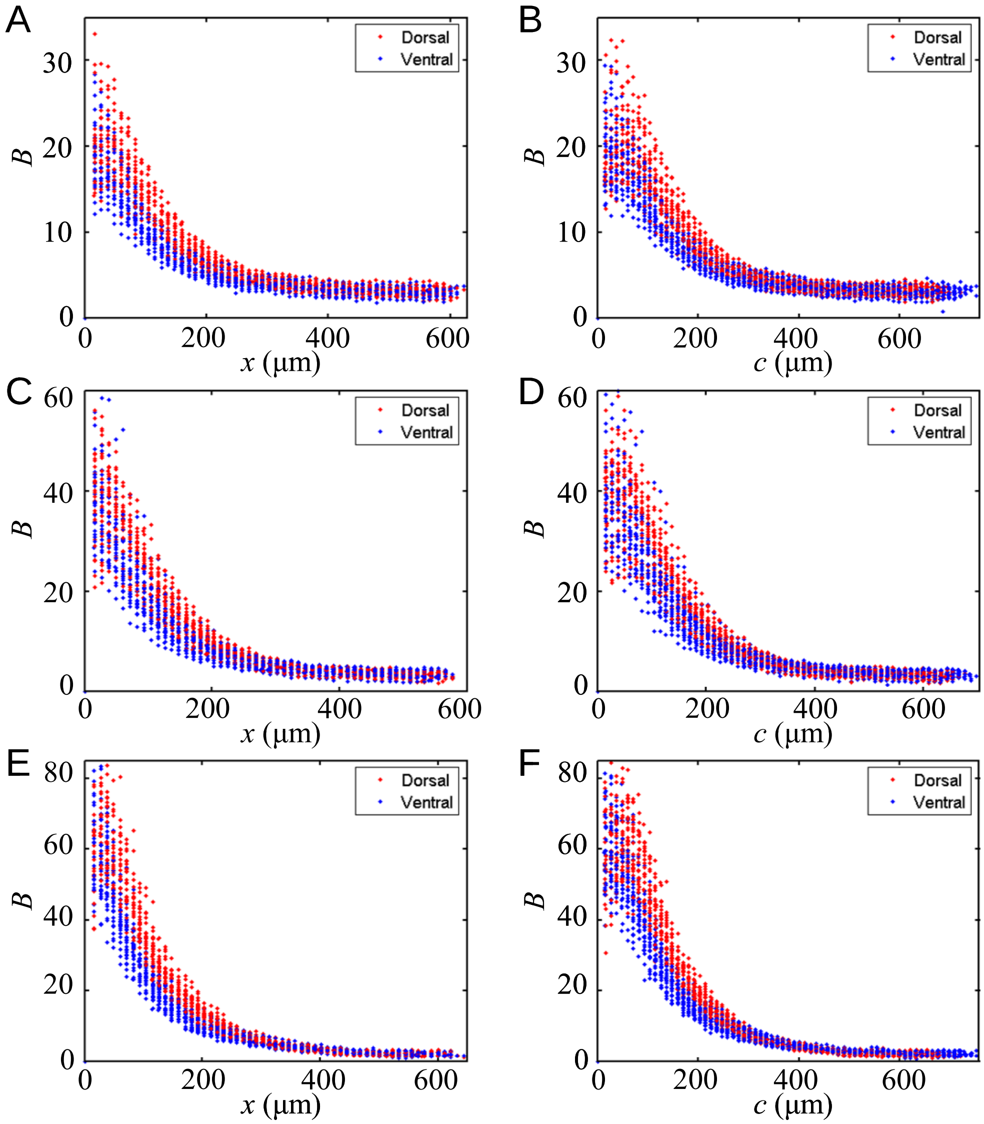

Supplement: Additional file 5 — Figure S4: Raw Bcd intensity data. (A and B) Raw Bcd intensity profiles on the dorsal (blue) and ventral (red) sides of 24 1×-bcd embryos when measured as a function of projected distance x from the anterior (A) or contour distance c (B). (C and D) Same as panels A and B, except the data are from 28 wt embryos. (E and F) Same as panels A and B, except the data are from 29 3×-bcd embryos. [file 1471-213X-10-80-S5.PDF]
