# Supplementary material for: Distance measurements via the morphogen gradient of Bicoid in Drosophila embryos
Source: BMC Dev Biol. 2010 Aug 2;10:80. doi: 10.1186/1471-213X-10-80 (PMC2919471; doi:10.1186/1471-213X-10-80)
Supplement: Additional file 7 — Figure S6: Simulating an embryo that has an asymmetric geometry. (A) A heat map of local total Bcd concentration on the midsagittal plane of a simulated embryo, which is approximated by two semi-ellipsoids joined on the coronal plane, with a height of 92 μm and 128 μm for the dorsal and ventral sides, respectively, and a length of 560 μm. For consistency, all other parameters here are set the same as those used to generate the results shown in Fig. 5. Color bar represents normalized local total Bcd concentration. (B and C) Heat maps of local total Bcd concentration of the same simulated embryo on transverse planes at xOtd/L (panel B) and xHb/L (panel C). Color bar represents normalized local total Bcd concentration only showing the range for the cortical layer. (D and E) Nuclear Bcd concentration profiles from the cortical layer of the dorsal (blue) and ventral (red) sides of the simulated embryo, measured as a function of either projected distance x from the anterior (panel D) or contour distance c (panel E). [file 1471-213X-10-80-S7.PDF]

Additional File 7

Figure S6. Simulating an embryo that has an asymmetric geometry

(A) A heat map of local total Bcd concentration on the midsagittal plane of a simulated embryo, which is approximated by two semi-ellipsoids joined on the coronal plane, with a height of 92 μm and 128 μm for the dorsal and ventral sides, respectively, and a length of 560 μm. For consistency, all other parameters here are set the same as those used to generate the results shown in Fig. 5. Color bar represents normalized local total Bcd concentration.

(B and C) Heat maps of local total Bcd concentration of the same simulated embryo on transverse planes at x_{Otd}/L (panel B) and x_{Hb}/L (panel C). Color bar represents normalized local total Bcd concentration only showing the range for the cortical layer.

(D and E) Nuclear Bcd concentration profiles from the cortical layer of the dorsal (blue) and ventral (red) sides of the simulated embryo, measured as a function of either projected distance x from the anterior (panel D) or contour distance c (panel E).

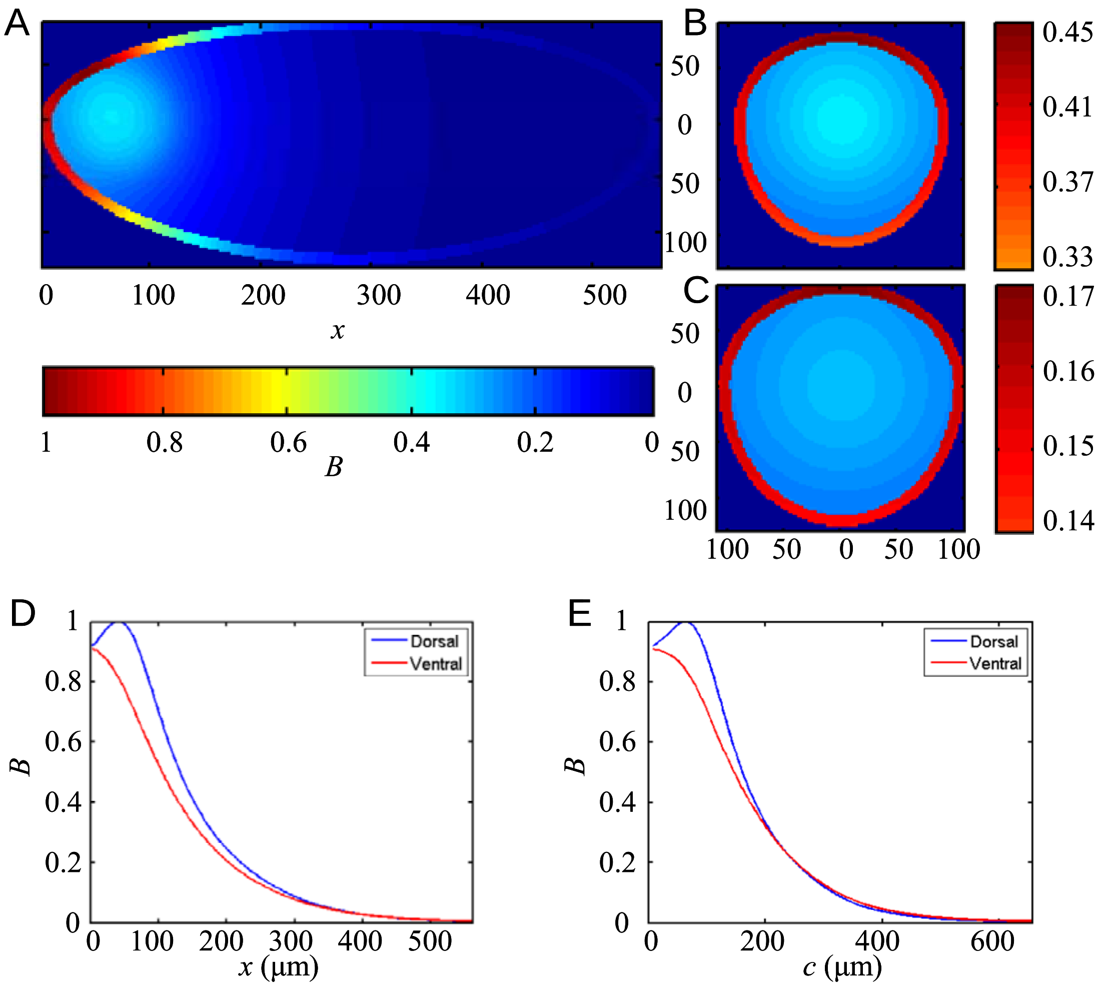

A
B
C
D
E
0
100
200
300
400
500
x
1
0.8
0.6
0.4
0.2
0
B
50
0
50
100
100 50 0 50 100
0.45
0.41
0.37
0.33
0.17
0.16
0.15
0.14
Dorsal
Ventral
x (μm)
c (μm)
600
