# Supplementary material for: Distance measurements via the morphogen gradient of Bicoid in Drosophila embryos
Source: BMC Dev Biol. 2010 Aug 2;10:80. doi: 10.1186/1471-213X-10-80 (PMC2919471; doi:10.1186/1471-213X-10-80)
Supplement: Additional file 8 — Figure S7: Testing the effects of bcd mRNA location and DNA binding on Bcd gradient properties. In these simulations, the center location of the bcd mRNA sphere was systematically moved either along the A-P axis (panels A-C; Rx denotes distance from anterior pole) or along the D-V axis (panels D-F; Ry denotes distance from the A-P axis). We also systematically changed the ratio of Bbound/Bfree at nuclear cycle 10 (panels G-I). Here, the shape of the simulated embryo is the same as in Fig. 5; for reference, the parameter values used in the simulations shown in Fig. 5 and Fig. S6 are marked with arrowheads. The resulting Bcd gradient profiles on the dorsal and ventral sides were then evaluated for their Bmax values (A, D and G), λ values expressed as either c or x (B, E and H), and the values of ΔxBcd and ΔcBcd at target boundary positions of both Hb and Otd (C, F and I). As shown in individual panels, these simulated results remain consistent with the three experimentally observed properties: 1) Bmax is higher on the dorsal side than on the ventral side (A, D and G), 2) λ measured as either x or c is larger on the ventral side than on the dorsal side (B, E and H; compare dashed lines with solid lines), and 3) ΔxBcd at target boundary locations is larger than their corresponding ΔcBcd (C, F and I; compare blue lines with red lines). As further discussed in the theoretical consideration above, the value of ΔxBcd - ΔcBcd in the middle section of the embryo reflects the geometric properties of the embryo. This value is 11.1 μm at xHb/L in our simulation results shown in this figure and, as expected, is insensitive to bcd mRNA location or the Bbound/Bfree ratio. It is also fully consistent with both experimental (10.7 μm as shown in Fig. 4A) and theoretical values (11.7 μm as shown in Fig. 1C). [file 1471-213X-10-80-S8.PDF]

Additional File 8

Figure S7. Testing the effects of *bcd* mRNA location and DNA binding on Bcd gradient properties.

In these simulations, the center location of the *bcd* mRNA sphere was systematically moved either along the A-P axis (panels A-C; Rx denotes distance from anterior pole) or along the D-V axis (panels D-F; Ry denotes distance from the A-P axis). We also systematically changed the ratio of B_{bound}/B_{free} at nuclear cycle 10 (panels G-I). Here, the shape of the simulated embryo is the same as in Fig. 5; for reference, the parameter values used in the simulations shown in Fig. 5 and Fig. S6 are marked with arrowheads. The resulting Bcd gradient profiles on the dorsal and ventral sides were then evaluated for their B_{max} values (A, D and G), λ values expressed as either c or x (B, E and H), and the values of Δx_{Bcd} and Δc_{Bcd} at target boundary positions of both Hb and Otd (C, F and I). As shown in individual panels, these simulated results remain consistent with the three experimentally observed properties: 1) B_{max} is higher on the dorsal side than on the ventral side (A, D and G), 2) λ measured as either x or c is larger on the ventral side than on the dorsal side (B, E and H; compare dashed lines with solid lines), and 3) Δx_{Bcd} at target boundary locations is larger than their corresponding Δc_{Bcd} (C, F and I; compare blue lines with red lines). As further discussed in the theoretical consideration above, the value of Δx_{Bcd} - Δc_{Bcd} in the middle section of the embryo reflects the geometric properties of the embryo. This value is 11.1 μm at x_{Hb}/L in our simulation results shown in this figure and, as expected, is insensitive to *bcd* mRNA location or the B_{bound}/B_{free} ratio. It is also fully consistent with both experimental (10.7 μm as shown in Fig. 4A) and theoretical values (11.7 μm as shown in Fig. 1C).

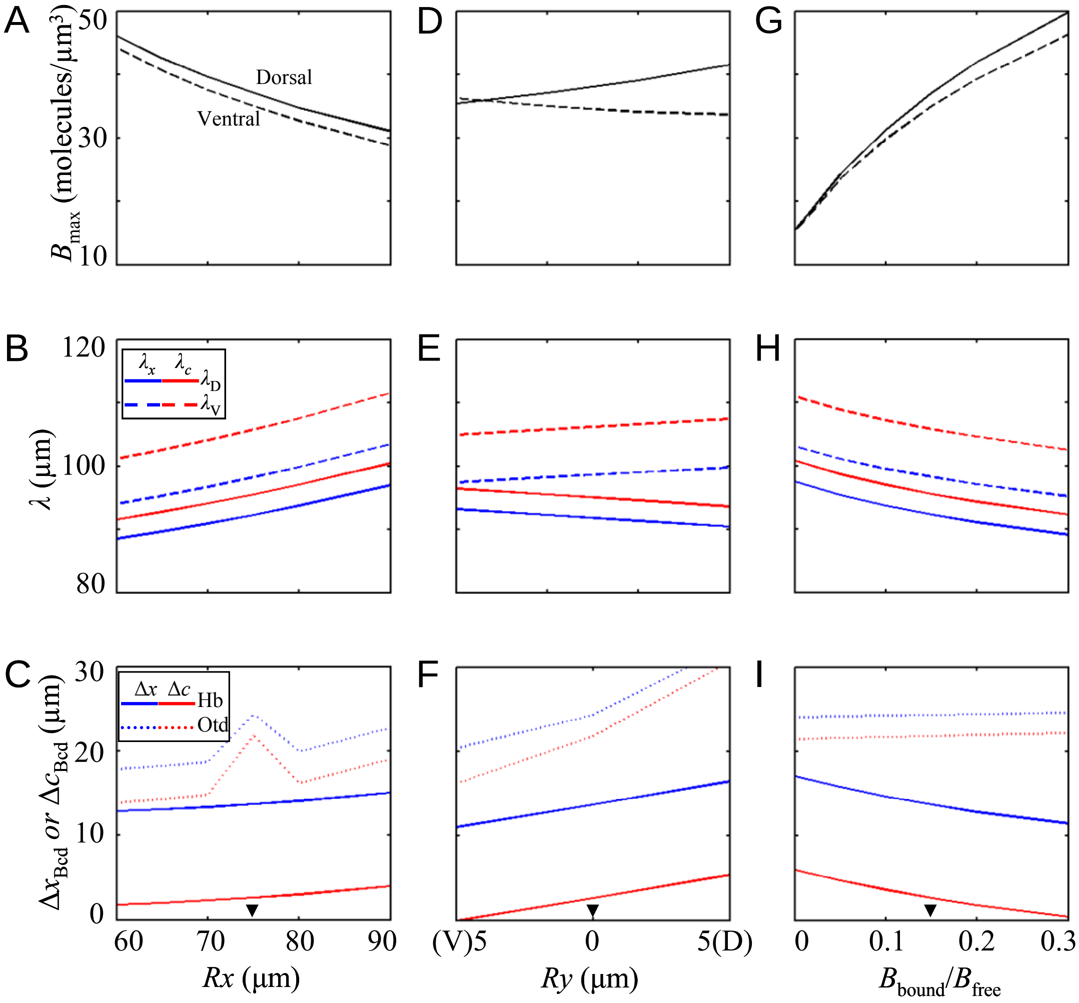

A
D
G
B
E
H
C
F
I
Dorsal
Ventral
B_{max} (molecules/μm^3)
50
30
10
120
100
80
λ (μm)
λ_x λ_c λ_D
λ_V
30
20
10
0
Δx_{Bcd} or Δc_{Bcd} (μm)
Δx Δc Hb
Otd
60 70 80 90
Rx (μm)
(V)5 0 5(D)
Ry (μm)
0 0.1 0.2 0.3
B_{bound}/B_{free}
