# Supplementary material for: Distance measurements via the morphogen gradient of Bicoid in Drosophila embryos
Source: BMC Dev Biol. 2010 Aug 2;10:80. doi: 10.1186/1471-213X-10-80 (PMC2919471; doi:10.1186/1471-213X-10-80)
Supplement: Additional file 9 — Table S1: Experimentally determined parameter values for the Bcd-Hb relationship. [file 1471-213X-10-80-S9.PDF]

Additional File 9

Table S1. Experimentally determined parameter values for the Bcd-Hb relationship.

	K_d			n		
	D	V	p	D	V	p
1×-*bcd*	3.6 ± 0.8	3.4 ± 0.8	0.24	4.8 ± 2.3	4.5 ± 2.7	0.57
2×-*bcd*	5.0 ± 1.2	4.8 ± 1.2	0.53	5.1 ± 2.7	4.9 ± 2.7	0.73
3×-*bcd*	3.8 ± 0.7	3.9 ± 0.8	0.51	3.7 ± 1.3	3.5 ± 0.7	0.30

	x_{Hb} (μm)			c_{Hb} (μm)		
	D	V	p	D	V	p
1×-*bcd*	223.8 ± 9.9	196.7 ± 10.0	10^{-11}	261.8 ± 9.9	244.0 ± 11.8	10^{-6}
2×-*bcd*	248.7 ± 9.2	232.2 ± 9.1	10^{-8}	284.6 ± 11.8	279.1 ± 10.8	0.07
3×-*bcd*	296.3 ± 9.9	283.4 ± 10.0	10^{-4}	335.4 ± 9.9	335.9 ± 11.8	0.88
